# Complete Genome Sequence of Bacteriophage Eula, Isolated on Microbacterium foliorum

**DOI:** 10.1128/mra.00728-22

**Published:** 2022-09-26

**Authors:** Travis Cook, Sophie Raffan, Taylor Born, Chloe Breece, Isaac Chandler, Henry DiLeo, Allison Eudy, Kaitlyn Lyon, Christian Nicholson, Adilene Serrano, Madeline Sigmon, Sydney Stevenson, Karli Townsell, Grace Van Patten, Justin E. Leonard, D. Parks Collins

**Affiliations:** a Department of Natural Sciences, Mitchell Community College, Statesville, North Carolina, USA; Queens College CUNY

## Abstract

Eula is a lytic microbacteriophage extracted from a soil sample collected in Statesville, NC, and isolated on Microbacterium foliorum NRRL B-24224. The Eula double-stranded DNA genome is 41,379 bp, with 69 predicted protein-coding genes and 1 tRNA. Based on gene content similarity, Eula was assigned to bacteriophage cluster EB.

## ANNOUNCEMENT

Bacteriophages are old, diverse, and the most abundant biological entity on earth. With an estimated population of 10^31^ virions (1), phages are found in every ecosystem inhabited by bacteria, including marine, terrestrial, and even gastrointestinal. Thus, phages are important to nutrient cycling (2) and microbe evolution (3) and can be alternatives to antibiotic treatments (4). The Science Education Alliance—Phage Hunters Advancing Genomics and Evolutionary Science (SEA-PHAGES) program has contributed to bacteriophage discovery using *Actinobacteria*, including Microbacterium foliorum, as isolation hosts.

**FIG 1:**
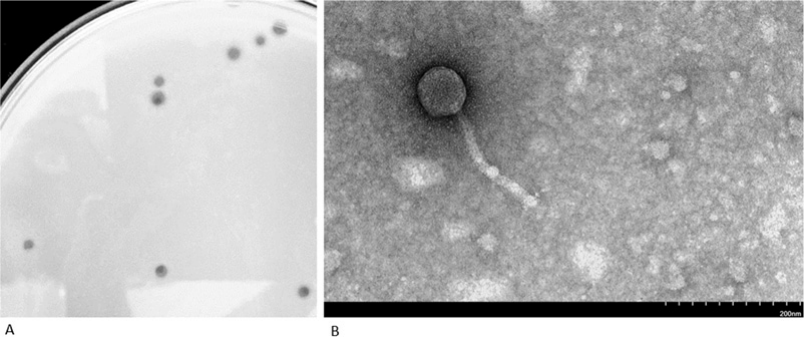
Plaques (A) and negative-stain transmission electron micrograph (B) of bacteriophage Eula.

Here, we report the genome sequence of bacteriophage Eula. Eula was collected from moist soil inside a chicken coop in Statesville, NC, USA (global positioning system [GPS] 35.799806 N, −80.890146 W). Following the standard procedures from the SEA-PHAGES *Phage Discovery Guide* (5), the soil sample was mixed with peptone-yeast extract-calcium (PYCa) liquid medium and incubated with shaking (200 rpm) for 2 h at 30°C. The mixture was then centrifuged and the supernatant filtered (pore size, 0.22 μm). The filtrate was inoculated with Microbacterium foliorum strain NRRL B-24224 and incubated for 48 h with shaking (220 rpm). A sample (1 mL) was then filtered (pore size, 0.22 μm), 10-fold serially diluted, and the dilutions plated in PYCa top agar with Microbacterium foliorum. Eula, which forms plaques with a clear center and turbid “halo” edges that are ~1.0 mm in diameter after 48 h at 30°C (Fig. 1A), was purified by three successive rounds of plating with *M. foliorum*. Negative-stain transmission electron microscopy revealed a *Siphoviridae* morphology (Fig. 1B) with a tail length of 177 to 188 nm and a capsid diameter of 68 to 74 nm (*n* = 3).

Double-stranded DNA was isolated from Eula using the Promega Wizard DNA cleanup kit (5). A sequencing library was prepared using the NEB Ultra II library kit and sequenced using an Illumina MiSeq instrument, yielding a total of 289,000 single-end 150-bp reads with 993× coverage. As described by Russell (6), the raw reads were assembled and checked for completeness using Newbler v2.9 (7) and Consed v29 (8), respectively. The linear genome comprises 51,375 bp with 10-nucleotide 3′ single-stranded cohesive ends (5′-TCTCCCGGCA-3′) and an average G+C content of 63.9%.

The Eula genome was autoannotated using DNA Master v5.23.6 (http://cobamide2.bio.pitt.edu/) embedded with Glimmer v3.02 (9) and GeneMark v2.5 (10) before the gene start sites were manually revised using Phamerator (11), Starterator v1.0.1 (https://seaphages.org/software), and PECAAN (https://blog.kbrinsgd.org/). Eula was predicted to contain 69 protein-coding genes and 1 tRNA coding for asparagine, identified using Aragorn v1.2.38 (12) and tRNAscan-SE v2.0 (13). Functional assignments were made using NCBI BLASTp (14) and HHpred (15), leading to putative functions for 32 out of 69 genes. Structural genes occupy the left third of the genome, while DNA metabolism genes occupy the right two-thirds. Eula lacks recognizable integrase or immunity repressor functions and is unlikely to establish lysogeny. Based on its gene content similarity of 35% or higher to genome sequences in the PhagesDB database (6, 16), Eula was assigned to bacteriophage subcluster EB. All tools were run with default parameters.

### Data availability.

Eula is available at GenBank under accession no. ON724018 and Sequence Read Archive (SRA) accession no. SRX14443500.
